# Spiritual and religious beliefs and behaviour: data collected from 27/28-year-old offspring in the Avon Longitudinal Study of Parents and Children, 2019-2020.

**DOI:** 10.12688/wellcomeopenres.17153.2

**Published:** 2024-04-08

**Authors:** Yasmin Iles-Caven, Iain Bickerstaffe, Kate Northstone, Jean Golding

**Affiliations:** 1 Bristol Medical School (Public Health Sciences), University of Bristol, Bristol, BS8 2BN, UK

**Keywords:** Religious belief, spirituality, religiosity, behaviour, ALSPAC, parent, offspring, sex differences

## Abstract

Religious/spiritual belief and practices have sometimes been demonstrated to have positive associations with outcomes such as coping with serious illness, anxiety, depression, negative life events and general well-being, and therefore warrants consideration in many facets of health research. For example, increasing secularisation evidenced, particularly in the West, may reflect increasing rates of depression and anxiety.

Very few studies have charted the ways in which religious/spiritual beliefs and practices of parents and their offspring vary longitudinally or between generations. Avon Longitudinal Study of Parents and Children (ALSPAC) is one such study that can relate belief and practices with aspects of physical and mental health and/or distinguish the different facets of the environment that may influence the development, or inter-generational loss, of belief and behaviours. This paper describes the 2019–2020 data collection in the ALSPAC on the religious/spiritual beliefs and behaviours (RSBB) of the study offspring (born 1991/1992) at ages 27–28 years. Previously collected and new data on the offspring are described here and comparisons are made with identical data completed by their parents (mothers and their partners) in early 2020.

The most striking observations are that in almost all aspects of RSBB the offspring of both sexes are more secular, especially when compared with their mothers. For example, 56.2% of offspring state that they do not believe in God, or a divine power compared with 26.6% of mothers and 45.3% of mothers’ partners. When asked about their type of religion, 65.4% of participants stated ‘none’, compared with 27.2% of mothers and 40.2% of partners. This confirms previous research reporting increasing secularisation from one generation to the next. As with the mothers and their partners, female offspring were more likely than males to believe in a divine power and to practice their beliefs.

## Introduction

Prior research has shown a steady decline in religious affiliation over time, dramatic increases in those stating they had no religion and a steady increase in non-Christian beliefs (e.g., Chaves, 2017; Office of National Statistics, 2012; The Pew Forum on Religion and Public Life, 2017). Younger generations demonstrate an increased tolerance of others’ beliefs, including non-belief (e.g., Curtice *et al.*, 2019). Most previous research has been cross-sectional and indicates that religious belief can be associated with positive health outcomes (see Koenig *et al.,* 2011), including reduced anxiety and depression ( Idler & Kasl, 1997). Increasing rates of depression and anxiety reported in younger generations (e.g., Collishaw *et al.,* 2009; Duffy *et al.,* 2019) may reflect the increasing secularisation evidenced particularly in the north-west of Europe, and increasingly, in the USA ( Chaves, 2017; Office of National Statistics, 2012; The Pew Forum on Religion and Public Life, 2017).

This paper describes the data concerning religious and spiritual beliefs and behaviours (RSBB) collected in 2019/2020 from the offspring (aged 27–28 years) of the original mothers enrolled during pregnancy in the Avon Longitudinal Study of Parents and Children (ALSPAC). Questions were designed to establish relationships with other longitudinal data from the cohort including traumatic incidents, physical and mental health, and genetic background. Research is planned to investigate various aspects of the antecedents and consequences of RSBB, and changes over time. A companion paper describes the RSBB data collected on the parents in 2020 ( Iles-Caven *et al.*, 2021).

 The data collected will be of importance in unravelling the current extent of influence of parents, peers, educational establishments, and organised religion on the beliefs and behaviours of the participants. As Hood and colleagues summarised (Chapter 5 pp 114–144), most research to date has been descriptive rather than explanatory ( Hood *et al.,* 2018). The data may also be used to assess genetic influences on RSBB which have been demonstrated previously in, for example, the Minnesota Twins study: whereby monozygotic and dizygotic twins who were brought up separately showed that 49% of the variation in religious measure scores appeared to be a function of heredity ( Waller *et al.*, 1990).

## Methods

### Participants

ALSPAC was specifically designed to determine ways in which the individual’s genotype combines with environmental pressures to influence health and development [ Golding *et al.*, 2001]. The study is geographically based in the south-west of England, centred around the city of Bristol and its surrounding rural and semi-urban areas, with a population of about one million. To capture as much valid information as possible, unbiased by knowledge of details of the characteristics of the baby, the study was designed to start as early in pregnancy as possible. All women resident in the area at the time they were pregnant were eligible, provided that their expected date of delivery lay between 1 ^st^ April 1991 and 31 ^st^ December 1992. Pregnant mothers (n=14,541), resident in the area, were recruited into the ALSPAC study. From these pregnancies, there were a total of 14,676 fetuses and 14,062 live births. Of the children, 13,988 were still alive at one year of age ( Boyd *et al.*, 2013).

Following advice from the ALSPAC Ethics and Law Committee, partners were recruited into the study *only* if the mothers wished them to be included. Questionnaires were sent to the mother who then passed the questionnaire on to the partner with a separate pre-paid return envelope. This method meant that the ALSPAC team were unable to follow up or communicate directly with the partners ( Birmingham, 2018; Fraser *et al.*, 2013). Therefore, the numbers of partners’ questionnaires returned were less than those received from the mothers. Around 75% of partners participated in the study. Partners were subsequently enrolled in their own right in 2010 (n=3000).

Major recruitment drives at the ages of seven and 18 years plus opportunistic contacts since age 7 enrolled additional eligible offspring. A total of 913 additional offspring participants have been enrolled in the study since the age of seven years with 195 of these joining since age 18. This additional enrolment provides a baseline sample of 14,901 offspring participants who were alive at one year of age. Of these 8964 were at known addresses, had not refused to take part, and were sent questionnaires ( Northstone *et al.*, 2019).

Since the offspring participants were aged 22 years, data have been collected and managed using REDCap electronic data capture tools hosted at the University of Bristol ( Harris *et al.*, 2009). REDCap (Research Electronic Data Capture) is a secure, web-based software platform designed to support data capture for research studies.

In summary, data have been collected from pregnancy onwards using a variety of methods: (a) self-completion questionnaires; (b) assays of biological samples; (c) hands-on examination of the subjects; (d) linkage to educational and health data on the individuals; (e) linkage of addresses to measures of geographic exposures; (f) information on schools attended with details of behaviour of the child and his/her parents completed by teachers and head teachers.

### Previous data collection on religious/spiritual beliefs and behaviour (RSBB) in the children

 Unlike the enrolled mothers and their partners who were asked about their RSBB on several occasions as described elsewhere ( Iles-Caven *et al.*, 2019; Iles-Caven *et al.*, 2021), only a few questions were asked of the mother concerning the child’s RSBB at the ages of 5, 9 and 11 years, and directly of the child at ages 16 and 18 years. Table 1 shows the frequency of responses to these questions. At age 5, frequency of Sunday School (a class held by a faith organisation) attendance was reported with 16% attending at least weekly; this compares with 7.8% at age 11. When asked whether the child took an interest in the meaning of life, there was a slight increase in ‘very interested’ and ‘somewhat interested’ between the ages of 9 and 11. This contrasted with those who took an interest in religion which decreased over the same period with a corresponding rise in the ‘no interest’ group from 32.2% to 40%. Similar decreases in worship attendance and praying are also evident. By age 11, 16.6% of mothers reported they were unsure whether their child prayed or not, compared with 8.8% at 9 years.

**Table 1.** Previous religious and spiritual beliefs and behaviours data collected on the participants during childhood (carer completed questionnaires ages 5, 9 and 11 years; offspring self-completed at 16 and 18 years).

Question	5 Years n (%)	9 Years n (%)	11 Years n (%)	16 Years n (%)	18 Years n (%)
*Frequency he/she attends Sunday School/other* * religious group during term time*					
1+/week	1437 (16.1)	-	-	-	-
Once a month	634 (7.1)	-	-	-	-
No, never	6843 (76.8)	-	-	-	-
*How often does he/she attend Sunday School?*					
2–7 times/week	-	19 (0.3)	18 (0.2)	-	-
Once a week	-	784 (9.7)	548 (7.6)	-	-
1–3 times/month	-	346 (4.3)	254 (3.5)	-	-
Less than 1/month	-	390 (4.8)	248 (3.4)	-	-
Never	-	6554 (81.0)	6184 (85.3)	-	-
*Does he/she take an interest in the meaning of life?*					
Yes, very interested	-	758 (9.5)	765 (10.6)	-	-
Somewhat interested	-	3510 (44.1)	3484 (48.2)	-	-
No, not interested	-	2862 (35.9)	2191 (30.3)	-	-
Not sure	-	832 (10.4)	782 (10.8)	-	-
*Does he/she take an interest in Religion?*					
Yes, very interested	-	785 (9.8)	567 (7.8)	-	-
Somewhat interested	-	4188 (52.1)	3231 (44.6)	-	-
No, not interested	-	2591 (32.2)	2895 (40.0)	-	-
Not sure	-	476 (5.9)	546 (7.5)	-	-
*Does he/she attend a place of worship?*					
Yes, often	-	1258 (16.2)	1000 (13.5)	-	-
Yes, sometimes	-	2298 (29.6)	1688 (22.8)	-	-
No, not at all	-	4206 (54.2)	4729 (63.8)	-	-
*Does he/she pray?*					
Yes, often	-	878 (11.2)	595 (8.1)	-	-
Yes, Sometimes	-	2763 (35.4)	1942 (26.4)	-	-
No, not at all	-	3482 (44.6)	3603 (48.9)	-	-
Not known	-	685 (8.8)	1226 (16.6)	-	-
*Has taken part in religious groups/organisations * *within school*					
Yes	-	-	-	185 (3.4)	-
No	-	-	-	5250 (96.6)	-
*Has taken part in religious groups/organisations * *outside school*					
Yes	-	-	-	392 (7.2)	-
No	-	-	-	5044 (92.8)	-
*Has attended a place of worship within last 4 weeks*					
Yes	-	-	-	-	456 (10.1)
No	-	-	-	-	4044 (89.9)

At age 16 the child was asked if they participated in religious groups/organisations both within school (3.4% did so) and outside of school (7.2% did so). By the age of 18 years, only 10% had attended a place of worship within the previous four weeks ( Table 1).

Table 2 describes the characteristics of those offspring participants who completed the RSBB questions. Women were more likely to complete the questionnaire than men, and the majority of respondents were educated to degree level. By 2020, many respondents were parents themselves (biological, step-parent, or adoptive); 21.7% were mothers and 12.5% were fathers. 15.6% still lived at home with their parents; 58.2% lived with a partner and 8.7% lived on their own.

**Table 2.** Selected demographics of the offspring participants responding to the 2019–2020 religious and spiritual beliefs and behaviours questions according to sex.

	Female N (%)	Male N (%)	Total N (%)
Age of their mother at birth of the offspring			
<25 years	430 (15.7)	188 (13.1)	618 (14.8)
25–34	1971 (72.0)	1045 (72.9)	3016 (72.3)
35+	337 (12.3)	200 (14.0)	537 (12.9)
Educational level *			
Any qualification	2266 (95.9)	1041 (95.2)	3307 (95.7)
Degree level qualification *			
Yes	1597 (67.5)	744 (68.1)	2341 (67.7)
No	768 (32.5)	341 (31.9)	1117 (32.3)
Parents lived together before they were born			
Yes	2490 (95.8)	1329 (97.2)	3819 (96.3)
No	108 (4.2)	38 (2.8)	146 (3.7)
Ethnic background			
White	2459 (96.1)	1314 (96.2)	3773 (96.2)
Other than white	99 (3.9)	52 (3.8)	151 (3.8)
Is currently a parent (biological, adopted, step etc)			
Yes	638 (21.7)	186 (12.5)	824 (18.6)
No	2302 (78.3)	1303 (87.5)	3605 (81.4)
Currently living			
on own			
Yes	237 (8.0)	151 (10.0)	388 (8.7)
No	2717 (92.0)	1359 (90.0)	4076 (91.3)
With children			
Yes	585 (19.8)	139 (9.2)	724 (16.4)
No	2369 (80.2)	1371 (90.8)	3740 (83.8)
With partner			
Yes	1821 (61.6)	778 (51.5)	2599 (58.2)
No	1134 (38.4)	732 (48.5)	1866 (14.8)
With parents			
Yes	410 (13.9)	288 (19.1)	698 (15.6)
No	2545 (86.1)	1222 (80.9)	3767 (84.4)
With friends/housemates			
Yes	371 (12.6)	244 (16.2)	615(13.8)
No	2584 (87.4)	1266 (83.8)	3850(86.2)
With family			
Yes	155 (5.2)	107 (7.1)	262 (5.9)
No	2800 (94.8)	1403 (92.9)	4203 (94.1)
With other			
Yes	41 (1.4)	40 (2.6)	81 (1.8)
No	2914 (98.6)	1470 (97.4)	4384 (98.2)

*Educational data collected at 26 years

### Data collection on RSBB 2019-2020

Identical questions on RSBB administered to their parents in 2020 were administered to the offspring at age 27–28 years in 2019–2020. The questionnaires were initially sent out in November 2019, 2770 were returned in 2019 and 1810 in 2020. 299 (6.5%) were returned after the Covid-19 lockdown in the UK, which took place on 23 ^rd^ March. The majority of responders (3801, 82.9%) completed the online version (the remainder (n= 783) returned a paper version through the post). The measures used are described in detail elsewhere ( Iles-Caven *et al.*, 2021), and comprised the original questions asked of the mothers and their partners over time, plus elements from well-validated, standardised scales (shown in bold in Table 3). In brief, these scales are:

(i) The **Duke University Religion Scale** (DUREL) ( Koenig *et al.*, 1997), a five-item measure of religious involvement developed for use in large studies. It assesses organisational and non-organisational religious activity (see Table 3 questions C9 and C11) and **intrinsic** religious motivation ( Table 3, questions C14-C16 when combined). The DUREL has been used extensively ( Koenig *et al.*, 2011).
(ii) Specific questions to elicit **extrinsic and intrinsic religious motivation** were included that had been adapted so they could be answered by non-believers ( Gorsuch & McPherson, 1989). We used two of the extrinsically weighted items in our questionnaires (see Table 3, questions C17 and C18).
(iii) Three questions (see Table 3, questions C19-C23) are from the well-validated **Fetzer Brief Multi-Dimensional Measure of Religiosity/Spirituality** for use in health research (BMMRS) ( Fetzer Institute & National Institute on Aging Working Group, 2003). These enquire whether an individual has had a religious/spiritual experience that changed their life or experienced a significant gain or loss of faith and if so at what age and to describe the event.

**Table 3.** Questions (numbered as in the questionnaire) asked of the participants in the 2019–2020 sweep, with their variable names. Items forming various validated scales are indicated in bold.

Questions 2019/2020	Variable name
*C1. Do you believe in God or in some divine power?* Yes/Not sure/No	*YPG3000*
*C2. Do you feel that God (or some divine power) has helped you at any time? * Yes/Not sure/No	*YPG3010*
*C3. Would you appeal to God for help if you were in trouble?* Yes/Not sure/No	*YPG3020*
*C4. Do you ‘pray’ even if not in trouble? * Yes/No	*YPG3030*
*C5. What sort of religious faith would you say you had? (tick only one)* None; Church of England; Roman Catholic; Jehovah’s Witness; Christian Science; Mormon; Other Christian (please describe); Jewish; Buddhist; Sikh; Hindu; Muslim; Rastafarian; Other (please describe)	*YPG3040*
*C6. How long have you had this particular faith?* All my life/More than 5 years/3-5 years/1-2 years/Less than a year	*YPG3050*
*C7. Were you brought up in this faith?* Yes/No/If no, please describe what faith if any	*YPG3060*
*C8. Would/Are you bringing your child(ren) up in your current faith/belief (including none)?* Yes this faith/No. If no, what faith did you bring your children up in, if any?	*YPG3070*
***C9. How often do you go to a place of worship or other religious meetings?*** Yes, at least once a week/Yes, at least once/month/Yes, at least once a year/Not at all	*YPG3080* ***DUREL***
*C10. Do you obtain help and support:*	
*-From leaders of your religious group? * Yes/No	*YPG3090*
*-From other members of your religious group?* Yes/No	*YPG3091*
*-From leaders of other religious groups (please describe)*? Yes/No	*YPG3092*
*-From members of other religious groups (please describe)*? Yes/No	*YPG3093*
***C11. How often do you spend time in private religious activities, such as prayer, meditation, or holy scripture study?*** More than once a day/Daily/2+times a week/Once a week/Few times a month/Rarely or never	*YPG3100* ***DUREL***
*C12. How often do you listen to/watch religious programming on the radio/ television/social media?* Daily/Several times a week/Several times a month/Occasionally/ Never/ Please describe	*YPG3110*
*C13. How often do you read religious related texts or publications (e.g. the Bible, the Koran, prayer book, Watchtower, The War Cry, The* * Friend, Spirituality & Health, Catholic Digest)* Daily/Several times a week/Several times a month/Occasionally/ Never/Please describe	*YPG3120*
***C14. In my life, I experience the Presence of the Divine (e.g. God)*** Definitely true of me/Tends to be true of me/Unsure/ Tends not to be true of me/Definitely not true of me/Not applicable	*YPG3130* ***DUREL Intrinsic***
***C15. My religious beliefs are what really lie behind my whole approach to life.*** Definitely true of me/Tends to be true of me/Unsure/Tends not to be true of me/Definitely not true of me/Not applicable	*YPG3140* ***DUREL*** ***Intrinsic***
***C16. I try hard to carry my religion over into all other dealings in life. *** Definitely true of me/Tends to be true of me/Unsure/Tends not to be true of me/Definitely not true of me/Not applicable	*YPG3150* ***DUREL*** ***Intrinsic***
***C17. I attend a place of worship mainly because it helps me make friends: *** Strongly agree/Mildly agree/Not sure/Mildly disagree/ Strongly disagree/Not applicable	*YPG3160* ***Extrinsic***
***C18. I pray mainly to gain relief and protection.*** *Strongly agree/Mildly agree/Not sure/Mildly disagree/Strongly disagree/Not applicable*	*YPG3170* ***Extrinsic***
***C19. Did you ever have a religious or spiritual experience that changed your life?*** Yes/No, If yes, age/please describe	*YPG3180* ***Fetzer***
***C20. Have you ever had a significant gain in your faith?*** Yes/No, If yes, age/please describe	*YPG3190* ***Fetzer***
***C21. Have you ever had a significant loss of faith? *** Yes/No, If yes, age/please describe	*YPG3200* ***Fetzer***
***C22. To what extent do you consider yourself a religious person?*** Very/Moderately/Slightly/Not at all	*YPG3210* ***Fetzer***
***C23. To what extent do you consider yourself a spiritual person?*** Very/Moderately/Slightly/Not at all	*YPG3220* ***Fetzer***
*C24. How important to you is religion or spirituality?* Highly/Moderately/Slightly/Not important at all	*YPG3230*

As can be seen from Table 4, young women were more likely than men to attend organised religious worship and to practice private worship (e.g., prayer). Female participants scored slighter higher means on the DUREL scale (4.55 vs. 4.24).

**Table 4.** Duke University Religion Scale (DUREL) derived variables. (P values are the probability that the responses for males and females are similar).

		Females n (%)	Males n (%)	Total n (%)	P value
	**Organised religion** ** activity score**				0.001
1	Not at all	2126 (72.9)	1164 (77.8)	3290 (74.7)	
2	Occasionally	424 (14.5)	155 (10.4)	589 (13.3)	
3	At least 1/yr	230 (7.9)	110 (7.4)	340 (7.7)	
4	At least 1/mth	46 (1.6)	26 (1.7)	72 (1.6)	
5	1+/week	92 (3.1)	41 (2.7)	133 (3.0)	
	**Private religious** ** activity score**				0.062
1	Rarely	2590 (88.7)	1358 (91.3)	3948 (89.6)	
2	Few/month	115 (3.9)	36 (2.4)	151 (3.4)	
3	1/wk	34 (1.2)	17 (1.1)	51 (1.2)	
4	2+/wk	69 (2.4)	36 (2.4)	105 (2.4)	
5	Daily	75 (2.6)	28 (1.9)	103 (2.3)	
6	>1/day	36 (1.2)	13 (0.9)	49 (1.1)	
	**Intrinsic score**				0.001
	Mean	4.55	4.24	4.45	
	SD	2.99	2.71	2.90	
	N	2925	1486	4411	
	**DUREL Total Index**				0.001
	Mean	7.35	6.90	7.20	
	SD	4.40	7.35	4.27	
	N	2880	1468	4348	

Table 5 shows the responses to each question by sex of the respondent. There is evidence to suggest that young women are more likely than the men to believe in a divine power, and more likely to participate in religious behaviours. This repeats the pattern we found for their mothers compared with the mothers’ partners ( Iles-Caven *et al.*, 2021).

**Table 5.** Responses to each question in 2019/2020 (P values are the probability that the responses for males and females are similar).

Question	Females n (%)	Males n (%)	P value
*Do you believe in God or some divine power?*			
Yes	533 (18.0)	223 (14.8)	<0.001
Not sure	854 (28.9)	343 (22.8)	
No	1567 (53.0)	941 (62.4)	
*Do you believe that God/divine power has helped you at any time?*			
Yes	511 (17.3)	197 (13.1)	<0.001
Not sure	593 (20.1)	236 (15.7)	
No	1845 (62.6)	1068 (71.2)	
*Would you appeal to God for help if you were in trouble?*			
Yes	707 (24.0)	246 (16.4)	0.003
Not sure	561 (19.0)	253 (16.8)	
No	1677 (57.0)	1005 (66.8)	
*Do you ‘pray’ even if not in trouble?*			
Yes	372 (12.6)	145 (9.7)	0.001
Not sure	207 (7.0)	88 (5.8)	
No	2368 (80.4)	1269 (84.5)	
*Would/Are you bringing your child(ren) up in your current faith/belief (including none)?* *If no, what faith did you bring your children up in, if any?*			
Yes, this faith	920 (47.1)	481 (49.5)	0.454
Not sure	668 (34.2)	318 (32.7)	
No	366 (18.7)	172 (17.7)	
*How long have you had this particular faith?*			
Whole life	2062 (72.9)	975 (66.7)	<0.001
>5 years	612 (21.6)	427 (29.2)	
3–5 years	80 (2.8)	32 (2.2)	
<3 years	76 (2.7)	28 (1.9)	
*Were you brought up in this faith?*			
*Yes/No/If no, please describe what faith if any*			
Yes, this faith	1717(60.2)	789(53.7)	<0.001
No	1137(39.8)	681(46.3)	
*Do you go to a place of worship?*			
At least once a week	92 (3.1)	41 (2.7)	0.001
At least once a month	46 (1.6)	26 (1.7)	
At least once a year	230 (7.9)	110 (7.4)	
Occasionally	434 (14.8)	155 (10.4)	
Never	2126 (72.6)	1164 (77.8)	
*Do you obtain help and support:*			
* From leaders of your religious group?*			
Yes	114 (3.9)	47 (3.2)	0.344
No	1528 (52.1)	746 (50.1)	
Not applicable *	1293 (44.1)	695 (46.7)	
*From members of your religious group?*			
Yes	151 (5.2)	63 (4.2)	0.297
No	1480 (50.6)	727 (48.8)	
Not applicable *	1292 (44.2)	701 (47.0)	
*From leaders of other religious group?*			
Yes	9 (0.3)	10 (0.7)	0.084
No	2780 (99.7)	1420 (99.3)	
*From members of other religious groups?*			
Yes	27 (1.0)	23 (1.6)	0.069
No	2750 (99.0)	1400 (98.4)	
*Type of religious belief*			
Stated “none”	1861 (63.5)	1032 (69.2)	0.001
Church of England	591 (20.2)	251 (16.8)	
Roman Catholic	122 (4.2)	39 (2.6)	
Other Christian (please describe) **	149 (5.1)	69 (4.6)	
Other non-Christian (please describe) ***	209 (7.1)	101 (6.8)	
***How often do you spend time in private religious activities, such as prayer, meditation, or holy*** *** scripture study?***			
More than once/day	36 (1.2)	13 (0.9)	0.062
Daily	75 (2.6)	28 (1.9)	
2+ times/week	69 (2.4)	36 (2.4)	
Once/week	34 (1.2)	17 (1.1)	
Few times/month	115 (3.9)	36 (2.4)	
Rarely or never	2590 (88.7)	1358 (91.3)	
***How often do you listen to/watch religious programming on the radio/ television/social media?***			
Daily	18 (0.6)	7 (0.5)	0.549
Several times/week	30 (1.0)	12 (0.8)	
Several times/month	23 (0.8)	18 (1.2)	
Occasionally	213 (7.3)	102 (6.8)	
Never	2651 (90.3)	1358 (90.7)	
***How often do you read religious related texts or publications (e.g. the Bible, the Koran, prayer book,*** *** Watchtower, The War Cry, The Friend, Spirituality & Health, Catholic Digest)***			
Daily	42 (1.4)	16 (1.1)	0.440
Several times/week	51 (1.7)	22 (1.5)	
Several times/month	32 (1.1)	21 (1.4)	
Occasionally	181 (6.2)	107 (7.1)	
Never	2630 (89.6)	1331 (88.6)	
***In my life, I experience the Presence of the Divine (e.g. God)***			
Definitely, true of me	117 (4.0)	52 (3.5)	<0.001
Tends to be true of me	153 (5.2)	50 (3.4)	
Unsure	316 (10.8)	131 (8.8)	
Tends not to be true of me	189 (6.4)	67 (4.5)	
Definitely, not true of me	977 (33.3)	625 (41.9)	
Not applicable *	1183 (40.3)	567 (38.0)	
***My religious beliefs are what really lie behind my whole approach to life***			
Definitely true of me	108 (3.7)	50 (3.4)	0.125
Tends to be true of me	157 (5.4)	68 (4.6)	
Unsure	201 (6.9)	89 (6.0)	
Tends not to be true of me	181 (6.2)	73 (4.9)	
Definitely not true of me	964 (32.9)	527 (35.4)	
Not applicable *	1322 (45.1)	682 (45.8)	
***I try hard to carry my religion over into all other dealings in life***			
Definitely true of me	87 (3.0)	43 (2.9)	<0.001
Tends to be true of me	160 (5.5)	55 (3.7)	
Unsure	168 (5.7)	56 (3.7)	
Tends not to be true of me	158 (5.4)	63 (4.2)	
Definitely not true of me	944 (32.2)	549 (36.7)	
Not applicable *	1411 (48.2)	728 (48.7)	
***I attend a place of worship mainly because it helps me make friends:***			
Strongly agree	37 (1.3)	13 (0.9)	0.253
Mildly agree	98 (3.3)	38 (2.5)	
Not sure	38 (1.3)	22 (1.5)	
Mildly disagree	78 (2.7)	30 (2.0)	
Strongly disagree	505 (17.2)	268 (18.0)	
Not applicable *	2175 (74.2)	1120 (75.1)	
***I pray mainly to gain relief and protection***			
Strongly agree	51 (1.7)	16 (1.1)	<0.001
Mildly agree	266 (9.1)	65 (4.4)	
Not sure	127 (4.4)	51 (3.4)	
Mildly disagree	87 (3.0)	37 (2.5)	
Strongly disagree	402 (13.8)	244 (16.4)	
Not applicable *	1986 (68.0)	1074 (72.2)	
***Did you ever have a religious or spiritual experience that changed your life?***			
Yes	140 (4.8)	74 (4.9)	0.792
No	2795 (95.2)	1421 (95.1)	
***Have you ever had a significant gain in your faith?***			
Yes	180 (6.1)	75 (5.0)	0.134
No	2751 (93.9)	1416 (95.0)	
***Have you ever had a significant loss of faith?***			
Yes	295 (10.1)	164 (11.0)	0.345
No	2637 (89.9)	1330 (89.0)	
***To what extent do you consider yourself a religious person?***			
Very	36 (1.2)	20 (1.3)	<0.001
Moderately	148 (5.0)	50 (3.3)	
Slightly	527 (17.9)	205 (13.7)	
Not at all	2227 (75.8)	1219 (81.6)	
***To what extent do you consider yourself a spiritual person?***			
Very	139 (4.7)	46 (3.1)	<0.001
Moderately	372 (12.7)	122 (8.2)	
Slightly	851 (29.0)	326 (21.9)	
Not at all	1575 (53.6)	997 (66.9)	
***How important to you is religion or spirituality?***			
Highly important	184 (6.3)	66 (4.4)	<0.001
Moderately important	274 (9.3)	121 (8.1)	
Slightly important	702 (23.9)	266 (17.8)	
Not important at all	1777 (60.5)	1038 (69.6)	

*P values are calculated excluding the ‘not applicable’ responses.

**Other Christian comprises: Christian Science, Mormon, Baptists, Evangelical, Methodists, Orthodox, Jehovah’s Witness etc.

***Other non-Christian comprises: Buddhism, Judaism, Sikhism, Hinduism, Muslim, Rastafarian, Spiritualism, New Age etc.

Comparisons of the responses to the RSBB questions of the offspring with those of their parents are shown in Table 6. Dramatic differences can be observed between them and their mothers. More than half of the offspring (56.2%) answering the question “Do you believe in God or some divine power?”, stated that they did not believe (compared with 26.6% of their mothers and 45.3% of partners). Responses to the question concerning type of religion indicate that 65.4% of offspring stated ‘none’, compared with 27.2% of their mothers and 40.2% of partners. 74.4% of offspring stated they never attend a place of worship compared with 49.8% of their mothers and 58.0% of their partners. In answer to the question “How important to you is religion or spirituality?”, 63.6% of offspring stated it was not important to them at all (compared with 39.2% of their mothers and 54.7% of the mothers’ partners). Slightly more offspring (5%) had had their current faith for ≤5years compared with their mothers (1.7%) or the mothers’ partners (1.6%). These results reflect previous research indicating sex differences in RSBB ( Fiori *et al.,* 2006; Coursey *et al.,* 2013) and increased secularism with each new generation (e.g., Twenge *et al.,* 2015).

**Table 6.** Comparison of beliefs and behaviours between offspring and parents in 2019/2020.

Question	Offspring (Total n=4580) n (%)	Mothers (Total n=4663) n (%)	Partners (Total n= 2181) n (%)
*Do you believe in God or some divine power?*			
Yes	758 (16.9)	2082 (43.5)	654 (30.0)
Not sure	1197 (26.8)	1429 (29.9)	538 (24.7)
No	2508 (56.2)	1270 (26.6)	986 (45.3)
*Do you believe that God/divine power has helped you at any time?*			
Yes	708 (15.9)	1651 (34.6)	509 (23.5)
Not sure	829 (18.6)	1222 (25.6)	424 (19.6)
No	2913 (65.5)	1897 (39.8)	1233 (56.9)
*Would you appeal to God for help if you were in trouble?*			
Yes	953 (21.4)	2319 (48.7)	670 (30.9)
Not sure	814 (18.3)	937 (19.7)	410 (18.9)
No	2682 (60.3)	1510 (31.7)	1089 (50.2)
*Do you ‘pray’ even if not in trouble?*			
Yes	517 (11.6)	1602 (33.8)	448 (20.7)
Not sure *	295 (6.6)	328 (6.9)	129 (6.0)
No	3637 (81.7)	2809 (59.3)	1588 (73.3)
*Did you bring your child(ren) up in your current faith/belief (including none)? If no, what faith* * did you bring your children up in, if any?(Parent)/ Were you brought up in this faith? (Offspring)*			
Yes, this faith	2506 (58.0)	3177 (67.6)	1335 (62.7)
No	1818 (42.0)	1524 (32.4)	794 (37.3)
*Are you/would you bring up your child(ren) in this faith? (offspring only)*			
Yes	1401 (31.7)	-	-
No	538 (12.2)	-	-
Not sure	986 (22.3)	-	-
Not applicable *	1490 (33.7)	-	-
*How long have you had this particular faith?*			
Whole life	3037 (70.8)	3467 (74.8)	1434 (67.8)
>5 years	1039 (24.2)	1091 (23.5)	649 (30.7)
3–5 years	112 (2.6)	46 (1.0)	23 (1.1)
<3 years	104 (2.4)	34 (0.7)	10 (0.5)
*Do you go to a place of worship?*			
At least once a week	133 (3.0)	423 (8.9)	168 (7.8)
At least once a month	72 (1.6)	205 (4.3)	83 (3.8)
At least once a year	340 (7.7)	359 (7.6)	162 (7.5)
Occasionally	589 (13.3)	1388 (29.3)	494 (22.9)
Never	3290 (74.4)	2359 (49.8)	1254 (58.0)
*Do you obtain help and support:*			
* From leaders of your religious group? *			
Yes	161 (3.6)	431 (9.2)	180 (8.4)
No	2274 (51.4)	3161 (67.1)	1303 (60.5)
Not applicable *	1988 (44.9)	1117 (23.7)	671 (31.2)
*Members of your religious group?*			
Yes	214 (4.8)	536 (11.5)	203 (9.5)
No	2207 (50.0)	3012 (64.7)	1254 (58.9)
Not applicable *	1993 (45.2)	1110 (23.8)	671 (31.5)
*From leaders of other religious groups?*			
Yes	19 (0.5)	68 (1.5)	31 (1.5)
No	4200 (99.5)	4373 (98.5)	2009 (98.5)
*Members of other religious groups?*			
Yes	50 (1.2)	110 (2.5)	46 (2.3)
No	4150 (98.8)	4283 (97.5)	1972 (97.7)
*Type of religious belief*			
Stated “none”	2893 (65.4)	1285 (27.2)	864 (40.2)
Church of England	842 (19.0)	2313 (48.9)	889 (41.4)
Roman Catholic	161 (3.6)	361 (7.6)	137 (6.4)
Methodist	41 (0.9)	182 (3.8)	57 (2.7)
Baptist/Evangelical	75 (1.7)	171 (3.6)	56 (2.6)
Other Christian (please describe) *	102 (2.3)	146 (3.1)	60 (2.8)
Judaism, Sikh, Hinduism, Muslim	32 (0.9)	27 (0.5)	14 (0.7)
Buddhist	27 (0.6)	34 (0.7)	17 (0.8)
Other non-Christian	251 (5.7)	213 (4.5)	53 (2.5)
*How often do you spend time in private religious activities, such as prayer, meditation, or holy* * scripture study?*			
More than once/day	49 (1.1)	130 (2.8)	60 (2.8)
Daily	103 (2.3)	354 (7.5)	100 (4.7)
2+ times/week	105 (2.4)	270 (5.7)	80 (3.8)
Once/week	51 (1.2)	129 (2.7)	38 (1.8)
Few times/month	151 (3.4)	287 (6.1)	84 (3.9)
Rarely or never	3948 (89.6)	3535 (75.1)	1768 (83.0)
*How often do you listen to/watch religious programming on the radio/ television/social media?*			
Daily	25 (0.6)	41 (0.9)	19 (0.9)
Several times/week	42 (0.9)	91 (1.9)	33 (1.5)
Several times/month	41 (0.9)	129 (2.7)	47 (2.2)
Occasionally	315 (7.1)	1421 (30.0)	556 (25.7)
Never	4009 (90.5)	3059 (64.5)	1511 (69.8)
*How often do you read religious related texts or publications (e.g. the Bible, the Koran, prayer* * book, Watchtower, The War Cry, The Friend, Spirituality & Health, Catholic Digest)*			
Daily	58 (1.3)	214 (4.5)	78 (3.6)
Several times/week	73 (1.6)	123 (2.6)	47 (2.2)
Several times/month	53 (1.2)	106 (2.2)	49 (2.3)
Occasionally	288 (6.5)	641 (13.5)	256 (11.8)
Never	3961 (89.4)	3656 (77.1)	1739 (80.2)
*In my life, I experience the Presence of the Divine (e.g. God)*			
Definitely, true of me	169 (3.8)	503 (10.7)	161 (7.5)
Tends to be true of me	203 (4.6)	507 (10.8)	156 (7.2)
Unsure	447 (10.1)	793 (16.9)	264 (12.2)
Tends not to be true of me	256 (5.8)	411 (8.7)	166 (7.7)
Definitely, not true of me	1602 (36.2)	1345 (28.6)	819 (37.9)
Not applicable *	1750 (39.5)	1146 (24.4)	593 (27.5)
*My religious beliefs are what really lie behind my whole approach to life*			
Definitely true of me	158 (3.6)	461 (9.8)	157 (7.3)
Tends to be true of me	225 (5.1)	723 (15.4)	257 (11.9)
Unsure	290 (6.6)	520 (11.1)	150 (7.0)
Tends not to be true of me	254 (5.7)	491 (10.5)	195 (9.0)
Definitely not true of me	1491 (33.7)	1256 (26.7)	693 (32.1)
Not applicable *	2004 (45.3)	1247 (26.5)	704 (32.7)
*I try hard to carry my religion over into all other dealings in life.*			
Definitely true of me	130 (2.9)	411 (8.8)	152 (7.1)
Tends to be true of me	215 (4.9)	667 (14.2)	213 (9.9)
Unsure	224 (5.1)	500 (10.7)	156 (7.2)
Tends not to be true of me	221 (5.0)	454 (9.7)	168 (7.8)
Definitely not true of me	1493 (33.8)	1296 (27.7)	702 (32.6)
Not applicable *	2139 (48.4)	1356 (28.9)	762 (35.4)
*I attend a place of worship mainly because it helps me make friends:*			
Strongly agree	50 (1.1)	87 (1.9)	18 (0.8)
Mildly agree	136 (3.1)	352 (7.5)	127 (5.9)
Not sure	60 (1.4)	155 (3.3)	80 (3.7)
Mildly disagree	108 (2.4)	309 (6.6)	123 (5.7)
Strongly disagree	773 (17.5)	939 (20.0)	406 (18.9)
Not applicable *	3295 (74.5)	2847 (60.7)	1399 (65.0)
*I pray mainly to gain relief and protection.*			
Strongly agree	67 (1.5)	212 (4.5)	39 (1.8)
Mildly agree	331 (7.5)	748 (16.0)	178 (8.3)
Not sure	178 (4.0)	388 (8.3)	123 (5.7)
Mildly disagree	124 (2.8)	351 (7.5)	146 (6.8)
Strongly disagree	646 (14.7)	740 (15.8)	369 (17.1)
Not applicable *	3060 (69.5)	2235 (47.8)	1297 (60.3)
*Did you ever have a religious or spiritual experience that changed your life?*			
Yes	214 (4.8)	542 (11.5)	196 (9.1)
No	4216 (95.2)	4171 (88.5)	1966 (90.9)
*Have you ever had a significant gain in your faith?*			
Yes	255 (5.8)	481 (10.3)	184 (8.6)
No	4167 (94.2)	4199 (89.7)	1967 (91.4)
*Have you ever had a significant loss of faith?*			
Yes	459 (10.4)	660 (14.1)	290 (13.5)
No	3967 (89.6)	4024 (85.9)	1864 (86.5)
*To what extent do you consider yourself a religious person?*			
Very	56 (1.3)	113 (2.4)	46 (2.1)
Moderately	198 (4.5)	653 (13.8)	255 (11.8)
Slightly	732 (16.5)	1549 (32.8)	512 (23.6)
Not at all	3446 (77.8)	2408 (51.0)	1352 (62.4)
*To what extent do you consider yourself a spiritual person?*			
Very	185 (4.2)	368 (7.8)	99 (4.6)
Moderately	494 (11.2)	937 (19.8)	347 (16.1)
Slightly	1177 (26.6)	1400 (29.6)	439 (20.3)
Not at all	2572 (58.1)	2019 (42.7)	1275 (59.0)
*How important to you is religion or spirituality?*			
Highly important	250 (5.6)	662 (14.0)	222 (10.2)
Moderately important	395 (8.9)	793 (16.8)	284 (13.1)
Slightly important	968 (21.9)	1417 (30.0)	476 (22.0)
Not important at all	2815 (63.6)	1853 (39.2)	1186 (54.7)

*Not sure/not applicable options added to 2019/2020 sweep only and did not appear in prior mothers/partners’ questionnaires.

## Strengths and limitations of the data

The strengths of these data include the large sample size, with ~4500 participants with data available from the 2019–2020 sweep, with comparable longitudinal RSBB data on their parents. The participants at birth were broadly representative of the general population in the area at the time of recruitment in terms of sex, ethnicity, and socio-economic status ( Fraser *et al.*, 2013).

A key limitation of the data is the lack of ethnic diversity. At the time of enrolment, the county of Avon was mainly Caucasian, therefore there were too few Black, Asian and Minority Ethnic (BAME) participants (<6% in the area) to allow for detailed analysis by ethnic background. Indeed, of the offspring participating in the 2020 sweep, <4% were non-white. The major limitation is that, as with all longitudinal studies there is increasing attrition over time, in particular males and those with lower levels of education were less likely to still be taking part in the study in their late twenties.

In this paper we do not advise on the appropriate techniques for accounting for missing data. Rather we have described the RSBB data collected on the study offspring so that interested researchers can use tools that they deem appropriate.

## Ethical approval and consent

Prior to commencement of the study, approval was sought from the ALSPAC Ethics and Law Committee and the Local Research Ethics Committees. Informed consent for the use of data collected via questionnaires and clinics was obtained from participants following the recommendations of the ALSPAC Ethics and Law Committee at the time. Questionnaires were completed in the participants own home and return of the questionnaires was taken as continued consent for their data to be included in the study. Full details of the approvals obtained are available from the study website. Study members have the right to withdraw their consent for elements of the study or from the study entirely at any time.

## Data Availability

ALSPAC data access is through a system of managed open access. The steps below highlight how to apply for access to the data included in this data note and all other ALSPAC data. Note that Table 3 in this paper gives the variable numbers for the recent sweep of religion data. Please read the ALSPAC access policy which describes the process of accessing the data and samples in detail, and outlines the costs associated with doing so. You may also find it useful to browse our fully searchable research proposals database, which lists all research projects that have been approved since April 2011. Please submit your research proposal for consideration by the ALSPAC Executive Committee. You will receive a response within 10 working days to advise you whether your proposal has been approved. If you have any questions about accessing data, please email alspac-data@bristol.ac.uk. The study website also contains details of all the data that is available through a fully searchable data dictionary.
