# Supplementary material for: Spiritual and religious beliefs and behaviour: data collected from 27/28-year-old offspring in the Avon Longitudinal Study of Parents and Children, 2019-2020
Source: Wellcome Open Res. 2024 Apr 8;6:215. Originally published 2021 Aug 24. [Version 2] doi: 10.12688/wellcomeopenres.17153.2 (PMC11190646; doi:10.12688/wellcomeopenres.17153.2)
Supplement: Peer review history

# Reviewer report: Reviewer response for version 2

**DOI:** 10.21956/wellcomeopenres.23188.r85798

**Published:** 2024-07-15

**Authors:** Margaret L Stuber

**Affiliations:** 1 University of California Los Angeles, Los Angeles, California, USA

Very interesting and novel set of data. Authors have responded to reviewers' comments. The results are complicate, given the time span of the study, and its multigenerational nature. The data are descriptive, with no real attempt to explain some of the dramatic differences between generations. It seems this article is to set up the data for future analyses.

Are sufficient details of methods and materials provided to allow replication by others?

Yes

Is the rationale for creating the dataset(s) clearly described?

Yes

Are the datasets clearly presented in a useable and accessible format?

Yes

Are the protocols appropriate and is the work technically sound?

Yes

Reviewer Expertise:

Childhood trauma

I confirm that I have read this submission and believe that I have an appropriate level of expertise to confirm that it is of an acceptable scientific standard.

# Reviewer report: Reviewer response for version 2

**DOI:** 10.21956/wellcomeopenres.23188.r84386

**Published:** 2024-06-20

**Authors:** Rodrigo Toniol

**Affiliations:** 1 University of Rio de Janeiro, Rio de Janeiro, Brazil

This article discusses spiritual and religious beliefs and behaviors in young adults. It discusses how these beliefs have changed over time and examines how the beliefs of young adults differ from those of their parents. The study found that young adults are more likely to be non-religious than their parents. They are also less likely to attend religious services or pray. For the authors, these findings suggest a trend toward secularization in Western societies.

 The text is a testament to the author's meticulous research and robust methodology, which is evident in the alignment of its conclusions with the data obtained. While the extensive discussion in the social sciences about the process of secularization in Western societies could have been a valuable addition to the article, I acknowledge that it falls outside the scope of the authors' area. Nevertheless, this text is poised to make a significant contribution to future discussions on secularization, bolstered by its rigorous research and compelling findings.

Are sufficient details of methods and materials provided to allow replication by others?

Yes

Is the rationale for creating the dataset(s) clearly described?

Yes

Are the datasets clearly presented in a useable and accessible format?

Yes

Are the protocols appropriate and is the work technically sound?

Yes

Reviewer Expertise:

Anthropology of religion

I confirm that I have read this submission and believe that I have an appropriate level of expertise to confirm that it is of an acceptable scientific standard.

# Reviewer report: Reviewer response for version 1

**DOI:** 10.21956/wellcomeopenres.18946.r52027

**Published:** 2022-09-14

**Authors:** Priya Wickramaratne

**Affiliations:** 1 Department of Psychiatry, Vagelos College of Physicians and Surgeons, Columbia University, New York, NY, USA

The effect of Religious and Spiritual Believe and Behavior (RSBB) has been shown to sometimes have positive associations with both physical and mental outcomes. 

 Very few studies have examined how parents and offspring RSBB vary between generations and over time. This study is one of the few existing studies that has the potential to compare the RSBB's role of the offspring with those of their mother and mother's partner.

 As noted by the authors, while most studies on this topic are descriptive, the data collected here can be viewed as explanatory and used to assess the effects of parents, peers, and educational establishments on the beliefs and behaviors of the participants.

 A further strength of the study is the size of the sample - over 4500 offspring participated in this study in 2019/2020 when the offspring were 27/28 years old. There are half as many males as female offspring in the sample and it is unclear whether the sample consisted of fewer males who responded or whether equal proportions of males and females responded, but there were more females overall in the sample than males.

 The datasets appear to be clearly presented in a useable and accessible format, although I have not had direct access to the datasets.

 A limitation of the paper, besides the lack of ethnic diversity in the sample, is the lack of discussion regarding attrition over time. It is unclear what should be considered the core sample of offspring and how missing data should be handled. Not accounting for missing data could result in biased estimates.

 Also, there are many gaps in time over the longitudinal data. Have the authors considered imputing the missing data?

Are sufficient details of methods and materials provided to allow replication by others?

Partly

Is the rationale for creating the dataset(s) clearly described?

Yes

Are the datasets clearly presented in a useable and accessible format?

Yes

Are the protocols appropriate and is the work technically sound?

Yes

Reviewer Expertise:

Psychiatry, Public Health and Biostatistics. Published several articles on religion.

I confirm that I have read this submission and believe that I have an appropriate level of expertise to confirm that it is of an acceptable scientific standard, however I have significant reservations, as outlined above.

# Response

**Published:** 2022-11-23

**Authors:** Yasmin Iles-Caven

**Affiliations:** University of Bristol, UK

Firstly, we would like to thank this reviewer for their useful comments, and we have responded to these:

*You wrote: ....A further strength of the study is the size of the sample - over 4500 offspring participated in this study in 2019/2020 when the offspring were 27/28 years old. There are half as many males as female offspring in the sample and it is unclear whether the sample consisted of fewer males who responded or whether equal proportions of males and females responded, but there were more females overall in the sample than males.*

 Response: The original births recruited to the ALSPAC study (and through subsequent recruitment phases) comprised 14,901 babies alive by age 1 year. Of these, 13,268 are still ‘active’ (i.e. they have not withdrawn), however, 8964 have agreed to be invited to complete questionnaires but not all do complete them (see Northstone et al., 2019 https://doi.org/10.12688%2Fwellcomeopenres.15132.1)

 As we point out in the Strengths & Limitations section, whilst the original babies were roughly 50/50 split on sex at birth, over the years it is the girls/women who are more likely to stay engaged with the study and hence the sex differences responding to the RSBB questionnaires at 27/28, with 65.8% of respondents being female. The study is also currently biased to higher education level, higher SES level and religious affiliation, but there are methods that could be used to overcome such biases such as multiple imputation or inverse-probability weighting.

*You wrote: A limitation of the paper, besides the lack of ethnic diversity in the sample, is the lack of discussion regarding attrition over time. It is unclear what should be considered the core sample of offspring and how missing data should be handled. Not accounting for missing data could result in biased estimates.*

 Response: The question as to what constitutes the core sample depends on the research question. Some authors who are interested in changes over time will use all those born to the women who were enrolled in the study; others will consider 8964 as the ‘core’ sample, to whom invitations to complete questionnaires are sent (slightly more have also indicated they would be interested in invitations to the face-to-face clinics). Roughly 50% of these complete questionnaires. This paper merely describes the data available, and it will be up to any researchers wishing to analyse the data how they handle missingness.

*You wrote: Also, there are many gaps in time over the longitudinal data. Have the authors considered imputing the missing data?*

 Response: Age 27/28 has been the only occasion that the G1 cohort (the offspring) have had as detailed information collected on them as on their parents (G0). Whilst one can compare the offspring during childhood with their parents’ RSBB at a similar time point, one cannot really fill in the missing data on RSBB, but again this would be up to the any researchers using this data to use the most suitable method(s) (e.g. imputation) for their analyses.

# Reviewer report: Reviewer response for version 1

**DOI:** 10.21956/wellcomeopenres.18946.r45638

**Published:** 2021-09-20

**Authors:** Peter Hill

**Affiliations:** 1 Rosemead School of Psychology, Biola University, La Mirada, CA, USA

The obvious value of this data set is its size, longitudinal nature, and the fact that it involves multiple respondents (offspring, mothers, mothers' partners) for comparison purposes. This report further documents what has already been established in the literature that 1) the younger generation is more secularized than both mothers and mothers' partners, 2) females are more religious than males, and 3) children become more disaffiliated with religion from the time they are in elementary school to the time of their adolescence, but does so based on a rich data set that, despite its ethnic and geographic limitations, is unmatched in terms of longitudinal longevity than any other data set available. Furthermore, the measures used for the data are diverse and cover many constructs that make the data valuable for other researchers to mine. The data set is clearly presented.

Are sufficient details of methods and materials provided to allow replication by others?

Yes

Is the rationale for creating the dataset(s) clearly described?

Yes

Are the datasets clearly presented in a useable and accessible format?

Yes

Are the protocols appropriate and is the work technically sound?

Yes

Reviewer Expertise:

Psychology religion and spirituality, especially measurement

I confirm that I have read this submission and believe that I have an appropriate level of expertise to confirm that it is of an acceptable scientific standard.

# Response

**Published:** 2022-11-23

**Authors:** Yasmin Iles-Caven

**Affiliations:** University of Bristol, UK

We thank the reviewer for taking the time to read and comment on our paper.

# Response

**Published:** 2024-01-25

**Authors:** Yasmin Iles-Caven

**Affiliations:** University of Bristol, UK

We thank the reviewer for taking the time to read and comment on our paper.
